# Large-Cell Esophageal Neuroendocrine Carcinoma: Report of a Rare Case

**DOI:** 10.7759/cureus.22041

**Published:** 2022-02-08

**Authors:** Ilias Galanis, Magdalini Simou, Georgios Floros

**Affiliations:** 1 2nd Department of Surgery, Evaggelismos General Hospital, Athens, GRC; 2 2nd Department of Surgery, Evangelismos General Hospital, Athens, GRC

**Keywords:** esophagogastrectomy, esophageal cancer, large cell neuroendocrine carcinoma, neuroendocrine tumor, esophagus

## Abstract

Neuroendocrine tumors (NETs) are neoplasms with neuroendocrine characteristics such as secretion of neuropeptides, large secretory vesicles, and a lack of neural structures. Neuroendocrine carcinoma (NEC) of the esophagus is a very rare malignancy. We present the case of a 58-year-old male with a pure large cell NEC of the esophagus.

## Introduction

Neuroendocrine tumors (NETs) are tumors developing from the enterochromaffin cells in the neuroendocrine tissue throughout the body [1]. Digestive system NETs have been classified by the World Health Organisation (WHO) in 2010 into three main categories: low grade (G1) NETs, intermediate (G2) NETs, and high grade (G3) carcinomas (neuroendocrine carcinomas [NECs]). NECs present a mitotic count of >20 per 10 high power fields (HPF) and/or a Ki-67 index of >20% [2].

NETs of the esophagus are very rare. This happens because the neuroendocrine system is not well developed in the esophagus. They represent only 1.3% of all the gastrointestinal tract NETs and 3.3% of all esophageal malignancies. Most of them, about 70%, are carcinomas [3]. The mean age of patients with esophageal NEC is 58.4 ± 8.2 years, with a 2.5/1 male predominance [2]. The majority of neuroendocrine carcinomas of the esophagus are small-cell NECs, while there are only a few cases of large-cell NECs, as in our case. Due to the rarity of these neoplasms, our knowledge of them is not well established, and further studies should be conducted to validate the best methods for diagnosing, staging, and treating them.

## Case presentation

**Figure 1:**
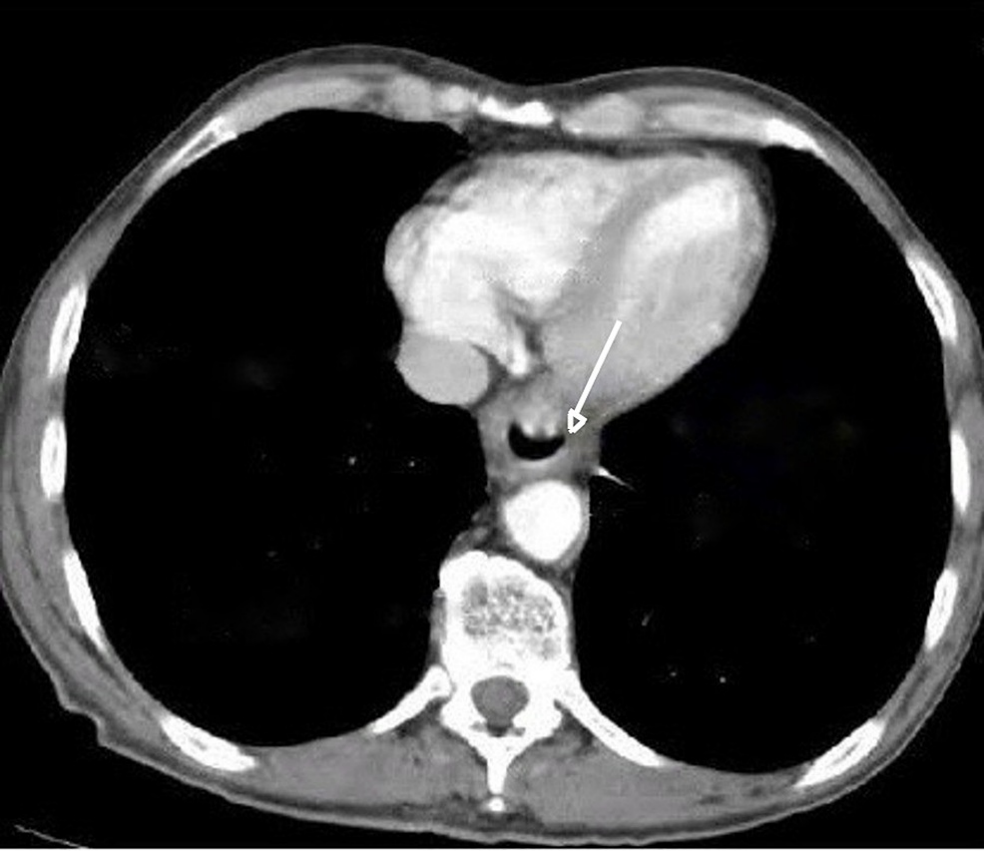
CT image showing a thickening of the wall of the lower esophagus

**Figure 2:**
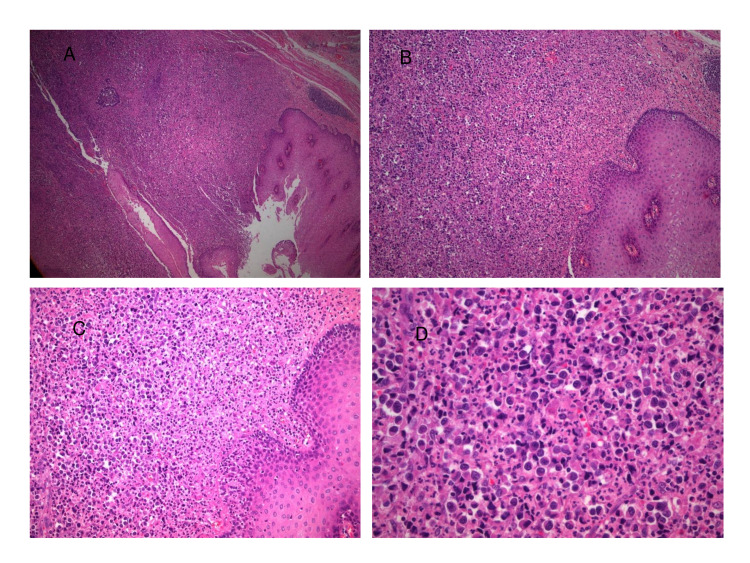
Infiltration of esophageal epithelium from a high grade/poorly cohesive-poorly differentiated large-cell carcinoma. (A) Hematoxylin-eosin ×40, (B) hematoxylin-eosin ×100, (C) hematoxylin-eosin ×200, and (D) hematoxylin-eosin ×200.

**Figure 3:**
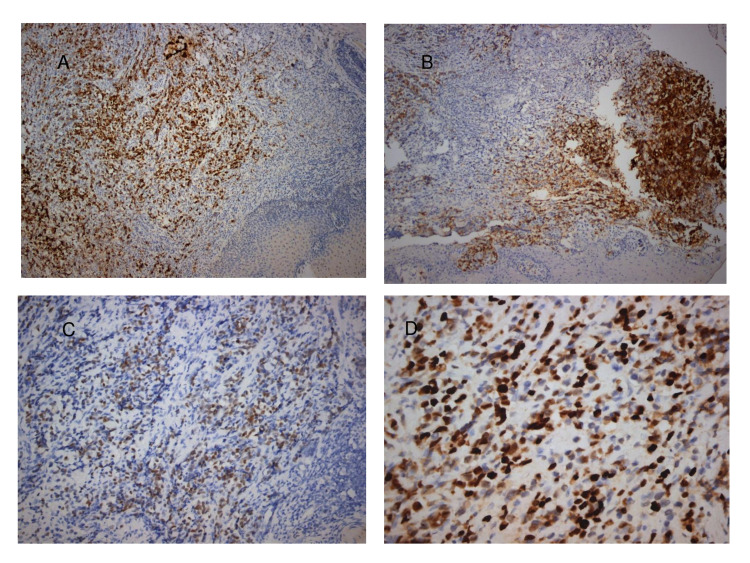
Immunophenotypical features of gastro-esophageal G3 neuroendocrine carcinoma. (A,B) Expression of neuroendocrine markers Chromogranin and Synaptophysin (×100), (C) expression of transcription factor CDX2 (×200), (D) Ki-67 proliferation marker is expressed in >70% of malignant cells.

A 58-year-old male presented to our surgical department with symptoms of dysphagia and abdominal discomfort for the past six months. The other past medical history was unremarkable. A computed tomography (CT) showed a 2 cm thickening of the wall of the lower esophagus without showing lymph node involvement (Figure 1). Esophagogastroduodenoscopy, which was performed, demonstrated an ulcerated mass of the distal esophagus expanding to the gastric cardia. Biopsies of the tumor led to the initial diagnosis of adenocarcinoma. A transhiatal esophagogastrectomy (Orringer technique), with an abdominal and a left neck incision, and a feeding jejunostomy were performed. A 2 × 1.5 mass was discovered in the lower esophagus and gastric cardia, without extending to the adventitia. The mass was grey/white, solid, with ulcerated areas on it. Microscopic examination showed large tumor cells with abundant eosinophilic cytoplasm, large nuclei with conspicuous eosinophilic nucleoli, and numerous mitoses (32 per 10 high power fields; Figure 2). Immunohistochemical studies demonstrated positive staining of all the cells for chromogranin and synaptophysin, putting the diagnosis of a G3 large cell NEC of the lower third of the esophagus into perspective. Ki-67 was >70% in tumor cells (Figure 3). The neoplasm infiltrated the muscularis propria without spreading to the periesophageal connective tissue. Metastasis was found in 1 out of 10 regional lymph nodes. The stage of the tumor was pT2N1, according to WHO2010/AJCC. On the eighth postoperative day, leakage from the cervical anastomosis was observed. Conservative management was decided with gradual refeeding and dilations. The patient underwent six cycles of adjuvant chemotherapy (cisplatin and etoposide), which has been proved to improve long-term survival following surgical resection of esophageal cancer, and on follow-up, there is no sign of recurrence, almost three years after the initial surgery.

## Discussion

McKeown first described NECs of the esophagus in 1952. Most of them are located in the mid to lower esophagus [3,4]. This is probably related to the endocrine cells in the esophageal cardiac glands that predominate in the distal region and the Merkel cells that exist in the middle esophagus. They are divided into two categories according to the levels of hormone secretion they produce: functional and non-functional. Despite the fact that esophageal NECs rarely secrete hormones, a small number of them may be identified as hormone-related syndromes, such as antidiuretic hormone secretion disorder syndrome [5]. These carcinomas are, typically, discovered as polypoid masses causing obstructive symptoms. They present with ulcerated necrosis and hemorrhage on the surface of the tumor. Their main symptoms are similar to those of other esophageal cancers: dysphagia, which is the most common, chest or epigastric discomfort, and weight loss [6]. Although the risk factors for esophageal NETs are not well defined, they seem to be the same as for squamous cell carcinoma, mainly smoking and alcohol consumption [7].

According to their histological characteristics, NECs of the esophagus are divided into two types: small-cell NECs and large-cell NECs, each of which possesses different morphological features. The cells in large-cell NECs, which are extremely rare, are quite large with prominent nucleoli and coarse chromatin, while in small-cell NECs they are small, containing inconspicuous nucleoli and fine granular chromatin [3,8]. Some esophageal cancers, especially large-cell NECs, include a neuroendocrine and an epithelial component (MiNENs). It is reported that the prognosis of these tumors is better than that of pure NECs [8]. The immunohistochemical profile of NECs is characteristic and helpful for the diagnosis of these neoplasms. NEC tumors display almost 100% positive staining for Synaptophysin, which is the most sensitive marker, and about 53% positive for Chromogranin-A. They are negative for p63, p40, CK5/6, and napsin A, which are mainly positive in squamous carcinomas and adenocarcinomas [9].

The grading of neuroendocrine neoplasms is based on histology and proliferative activity. As a result, there are grade 1 (low grade) neoplasms (<2 mitotic figures/10 HPF and 2% or less Ki-67 positive), grade 2 (intermediate grade, with 2-20 mitoses/10 HPF) and grade 3 (high grade) neoplasms (>20 mitoses/10 HPF and >20% Ki-67 positive) [5]. Esophageal NETs are staged according to the TNM classification system for oesophageal squamous cell carcinomas [10]. According to the American Joint Committee on Cancer (AJCC), the new (2017) TNM classifications for esophageal cancer staging now include three separate classifications: the clinical (c) (before treatment decision), pathologic (p) (after esophagectomy alone), and postneoadjuvant (yp) ones. Historically, pathologic stage grouping after esophagectomy alone has been the cornerstone for all cancer staging. In the present era, pathologic staging is losing its clinical relevance for advanced-stage cancer patients as neoadjuvant therapy replaces esophagectomy alone. It remains, however, an important staging and survival reference tool for early-stage cancers. In the 2017 edition of the TNM classification, there is an important rearrangement of the staging groups for squamous cell carcinoma and, as a result, for esophageal NECs. pStage 0 is restricted to high-grade glandular dysplasia, pTis. Subcategorization of T1 combined with grade requires two pStage I subgroups: pStage IA (pT1aN0M0G1) and pStage IB (pT1aN0M0G2-3, pT1bN0M0, and pT2N0M0G1). pStage IIA comprises pT2N0M0G2-3 cancers, pT3N0M0 cancers of the lower thoracic esophagus, and pT3N0M0G1 cancers of the upper-middle thoracic esophagus. pStage IIB comprises T3N0M0G2-3 cancers of the upper-middle thoracic esophagus and pT1N1M0 cancers. pStage III and pStage IV are identical for both adenocarcinoma and squamous cell carcinoma. New to the eight edition is the stage grouping of patients with esophageal cancer who have undergone postneoadjuvant therapy. The groups are identical for all histopathologic cell types. The grade is not included in postneoadjuvant pathologic staging. ypStage I comprises ypT0-2N0M0 cancers. ypStage II consists of a single entity, ypT3N0M0. ypStage IIIA comprises cancers confined to the esophageal wall with ypN1 regional nodal category (ypT0-2N1M1). ypStage IIIB comprises ypT1-3N2M0, ypT3N1M0, and ypT4aN0M0 cancers. ypStage IVA includes ypT4aN1-2M0, ypT4bN0-2M0, and yp TanyN3M0. ypStage IVB comprises ypM1 cancers. Finally, also new is the clinical stage grouping, before treatment decision, which relies on imaging of the tumor and not microscopic examination. cStage 0 comprises cTis. cStage I consists exclusively of cT1N0-1M0. cStage II comprises cT2N0-1M0 and cT3N0M0 cancers. cStage III comprises cT3N1M0 and cT1-3N2M0 cancers. cT4N0-2M0 and all cN3M0 cancers are placed in cStage IVA. cStage IVB is reserved for cM1 cancers [11]. The most important prognostic factor for the survival of patients is the invasion depth of the tumor, as it has been reported that there is a major difference in prognosis between patients with T1 and T2-4 neoplasms [8]. A high frequency of lymph node and distant metastases has been noted for NECs of the esophagus, especially large cells, with the liver and the lung being the most common sites of metastasis [12].

Chromogranin A (CgA) is an acidic glycoprotein specifically expressed in neuroendocrine cells. There have been multiple indications of the efficacy of CgA as a potential biomarker for NECs. More specifically, serum CgA levels are higher in patients with NENs than in healthy individuals, especially in patients with carcinoid syndrome, and they are found to be significantly decreased as a result of clinical response. It has also been revealed that patients with high serum CgA levels have significantly poorer survival than those with low CgA levels. For these reasons, serum CgA levels have been accepted as a very good circulating biomarker for both the diagnosis and follow-up of patients with neuroendocrine tumors, including esophageal NETs [13].

Despite the fact that there is no established treatment regime for esophageal neuroendocrine tumors, surgical resection, when it is possible, and/or radiotherapy and adjuvant chemotherapy, is the treatment of choice. According to recent studies, patients treated with surgery have a median survival time of 10.1 to 28.5 months, while those who were treated with chemotherapy alone have a median survival time of 8 to 16.1 months [14]. There is currently no standard choice of chemotherapy. Chen et al. reported that radical surgery and postoperative chemotherapy exhibit better outcomes [15]. The most commonly used drugs were platinum-based two-drug combinations, which remain the most frequently used drugs so far. Krug et al. reported that a streptozotocin (STZ) based combination should be considered for moderately and well-differentiated tumors, while a platinum-based regimen should be considered for poorly differentiated NETs [16]. Other treatments, such as somatostatin analogs and interferons, which reduce hormone secretion and improve the clinical symptoms, have been used. Moreover, bevacizumab in combination with other chemotherapy may lead to improved outcomes [5]. However, all these data still have to be confirmed by further research.

## Conclusions

NECs of the esophagus are rare but aggressive neoplasms. Large-cell carcinomas are the rarest among them. Their frequency rises more and more, thanks to the progress of our diagnostic methods. More research has to be done to ameliorate our lack of knowledge of these tumors and to provide better treatment options for the patients.
